# Induction of long-term potentiation and depression in individual synapses of CA1 pyramidal neurons

**DOI:** 10.1186/1471-2202-16-S1-P164

**Published:** 2015-12-18

**Authors:** Rosanna Migliore, Giada De Simone, Michele Migliore

**Affiliations:** 1 Institute of Biophysics, National Research Council, via Ugo La Malfa 153, 90146 Palermo, Italy

## 

Long-Term Potentiation (LTP) and Depression (LTD), the two major forms of long-lasting synaptic plasticity in the mammalian neurons, represent a basic step to understand neuronal development, circuit reorganization, learning and memory mechanisms. Experimental studies on LTP and LTD are usually performed using in vitro or in vivo preparations, exploiting repetitive and properly patterned stimulation protocols to induce long-lasting changes in the strength of a synaptic connection. Although the underlying molecular mechanisms are starting to be unraveled and are under intense experimental and theoretical scrutiny, especially in the CA1 region of the hippocampus, experimenters often report problems in using standard induction protocols to obtain consistent results, especially for LTD in vivo. We hypothesize that a possible source of confusion in interpreting the results, from any given experiment on synaptic plasticity, can be the intrinsic limitation of the experimental techniques, which cannot take into account the actual state and peak conductance of the synapses before the conditioning protocol.

Within this context, using biophysical models of synaptic plasticity and hippocampal CA1 pyramidal neurons, we investigate the relation between what is observed at the soma and LTP/LTD induction patterns, pre-conditioning synaptic strength, and dendritic location.

Our model and the results pointed out that the outcome of an experiment, testing the amount of synaptic LTP/LTD plasticity that can be induced, strongly depends at least on the initial synaptic state and peak conductance. In addition, the model explains why LTD induction may be more critical to be obtained, with respect to LTP, especially *in vivo*, and suggests experimentally testable predictions on the stimulation protocols that may be more effective.

